# Multi-Scale Analysis and Testing of Tensile Behavior in Polymers with Randomly Oriented and Agglomerated Cellulose Nanofibers

**DOI:** 10.3390/nano10040700

**Published:** 2020-04-07

**Authors:** Fumio Narita, Yinli Wang, Hiroki Kurita, Masashi Suzuki

**Affiliations:** Department of Materials Processing, Graduate School of Engineering, Tohoku University, Sendai 980-8579, Japan; wang.yinli.r2@dc.tohoku.ac.jp (Y.W.); kurita@material.tohoku.ac.jp (H.K.); masashi.suzuki.t5@dc.tohoku.ac.jp (M.S.)

**Keywords:** multi-scale mechanics, finite element analysis, material testing, cellulose nanofiber, polymer composites, tensile modulus

## Abstract

Cellulose nanofiber (CNF) has been accepted as a valid nanofiller that can improve the mechanical properties of composite materials by mechanical and chemical methods. The purpose of this work is to numerically and experimentally evaluate the mechanical behavior of CNF-reinforced polymer composites under tensile loading. Finite element analysis (FEA) was conducted using a model for the representative volume element of CNF/epoxy composites to determine the effective Young’s modulus and the stress state within the composites. The possible random orientation of the CNFs was considered in the finite element model. Tensile tests were also conducted on the CNF/epoxy composites to identify the effect of CNFs on their tensile behavior. The numerical findings were then correlated with the test results. The present randomly oriented CNF/epoxy composite model provides a means for exploring the property interactions across different length scales.

## 1. Introduction

Cellulose nanofiber (CNF) has been accepted as a valid nanofiller that can improve the mechanical properties of composite materials by both mechanical and chemical methods [1,2,3,4]. Nishino et al. [5] determined that the elastic modulus of natural cellulose in the longitudinal direction was 138 GPa. Blefdzki et al. [6] proposed that the ultimate tensile strength of cellulose is estimated to be 17.8 GPa, which is seven times higher than that of steel. Sain and Oksman [7] studied the mechanical properties of CNF to design CNF-reinforced composite materials. Chirayil et al. [8] and Ansari et al. [9] showed that the mechanical properties of CNF-dispersed polymer composites increased compared with those of pure polymer. However, it was reported that an agglomeration of CNF in the matrix, poor adhesion, and water uptake decreased the mechanical properties. Kurita et al. [10] investigated the flexural properties of the CNF-dispersed epoxy layer inserted into woven glass fiber-reinforced polymer (GFRP) composite laminates. They indicated that the flexural strength increases, whereas the flexural modulus is almost constant when inserting two CNF/epoxy interface layers. Xie et al. [11] prepared extremely low CNF-reinforced epoxy matrix composites and tried to understand the strengthening mechanism of the CNF/epoxy composite through a three-point flexural test and finite element analysis (FEA). They proposed the possibility that CNF behaves as an auxiliary agent to enhance the structure of epoxy molecules and not as a reinforcing fiber in the epoxy matrix.

Prediction of the stress and strain requires a detailed FEA. The finite element method is regarded as an important tool in the design and analysis of composite materials, and some finite element works on the modeling of composite materials have been presented by our group [12,13,14]. 

In this paper, the tensile behavior of CNF-reinforced polymer composites is investigated. A finite element model that considered the randomly oriented CNF in the polymer was developed, and FEA was performed for the composites with randomly oriented CNFs. Tensile tests were then carried out on the CNF/epoxy composites. The stress–strain curve and Young’s modulus for the CNF-reinforced polymer composites were then discussed in detail.

## 2. Numerical Procedure

### 2.1. Definition of Representative Volume Element

**Figure 1:**
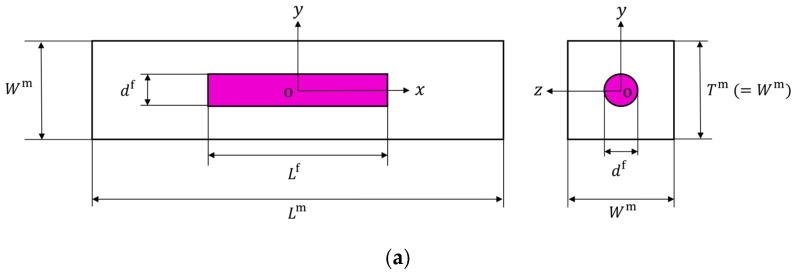

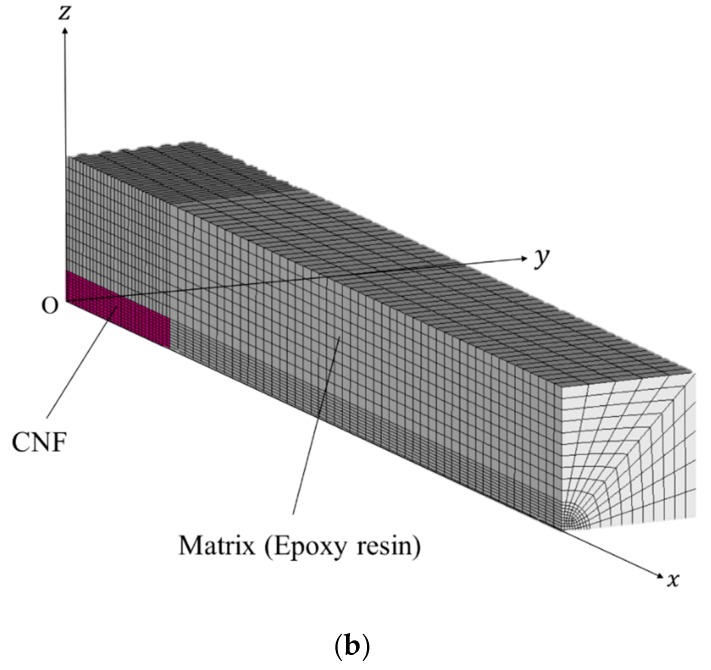
(**a**) Representative volume element (RVE) of cellulose nanofiber (CNF)-reinforced epoxy composite. (**b**) One-eighth finite element model of RVE for CNF-reinforced epoxy composite.

In this study, ANSYS Multiphysics code (ver. 11.0) was used to perform a three-dimensional FEA. Figure 1 shows the representative volume element (RVE) of the CNF-reinforced polymer composites. As shown in Figure 1a, the RVE was created such that the CNF was embedded in the center of the polymer matrix. The Cartesian coordinate system o-xyz was defined such that the x-axis was in the longitudinal direction, and the y- and z-axes were in the transverse plane of the RVE. $L^{f}$ and $L_{}^{m}$ represent the length of the CNF and matrix, respectively. The superscript f represents the fiber, and m denotes the matrix. $d_{}^{f}$ is the diameter of the CNF. $W_{}^{m}$ and $T_{}^{m}$ are the width and thickness of the matrix, respectively. Here, $T_{}^{m}$ was set to be equal to $W_{}^{m}$ so that the cross-section yz-plane is square. The CNF aspect ratio $L_{}^{f}$/$d_{}^{f}$ was assumed to be equal to the matrix aspect ratio $L_{}^{m}$/$W_{}^{m}$ [15], and the relationship between $W_{}^{m}$ and CNF volume fraction $V_{}^{f}$ was given by the following equation:

(1) $$V^{f}=\frac{\pi }{4}{\left(\frac{d^{f}}{W^{m}}\right)}^{3}$$

The CNF and matrix were assumed to have isotropic elastic properties, and the constitutive equation can be written as

(2) $$\left(\begin{array}{c} \varepsilon_{xx}^{\delta }\\ \varepsilon_{yy}^{\delta }\\ \varepsilon_{zz}^{\delta }\\ 2\varepsilon_{yz}^{\delta }\\ 2\varepsilon_{zx}^{\delta }\\ 2\varepsilon_{xy}^{\delta } \end{array}\right)=\left(\begin{array}{cccccc} 1/E^{\delta } & -\nu^{\delta }/E^{\delta } & -\nu^{\delta }/E^{\delta } & 0 & 0 & 0\\ -\nu^{\delta }/E^{\delta } & 1/E^{\delta } & -\nu^{\delta }/E^{\delta } & 0 & 0 & 0\\ -\nu^{\delta }/E^{\delta } & -\nu^{\delta }/E^{\delta } & 1/E^{\delta } & 0 & 0 & 0\\ 0 & 0 & 0 & 1/G^{\delta } & 0 & 0\\ 0 & 0 & 0 & 0 & 1/G^{\delta } & 0\\ 0 & 0 & 0 & 0 & 0 & 1/G^{\delta } \end{array}\right)\left(\begin{array}{c} \sigma_{xx}^{\delta }\\ \sigma_{yy}^{\delta }\\ \sigma_{zz}^{\delta }\\ \sigma_{yz}^{\delta }\\ \sigma_{zx}^{\delta }\\ \sigma_{xy}^{\delta } \end{array}\right)\left(\delta =m,\;f\right)$$

where $\varepsilon_{ij}^{\delta }$ and $\sigma_{ij}^{\delta }$ are the strain and stress components (i, j=x, y, z), $E_{}^{\delta }$ is the Young’s modulus, $G_{}^{\delta }$ is the shear modulus, and $\nu_{}^{\delta }$ is the Poisson’s ratio.

### 2.2. Finite Element Analysis of Representative Volume Element

The RVE of CNF-reinforced polymer composites can be assumed to be transversely isotropic with the plane of isotropy being the yz-plane (see Figure 1). The solution obtained from the analysis of the RVE is equivalent to the solution of unidirectional, uniformly dispersed, CNF-reinforced polymer composites. So, for the transversely isotropic RVE, there are five independent elastic properties: longitudinal Young’s modulus $E_{l}^{r}$, transverse Young’s modulus $E_{t}^{r}$, longitudinal Poisson’s ratio $\nu_{\mathrm{lt}}^{r}$, transverse Poisson’s ratio $\nu_{\mathrm{tt}}^{r}$, and longitudinal shear modulus $G_{\mathrm{lt}}^{r}=G_{\mathrm{tl}}^{r}$. The superscript r denotes the RVE. In addition, transverse shear modulus $G_{\mathrm{tt}}^{r}$ is calculated by $G_{\mathrm{tt}}^{r}=E_{t}^{r\;}/2\left(1+\nu_{\mathrm{tt}}^{r}\right)$. Then, the longitudinal Young’s modulus $E_{l}^{r}$ and Poisson’s ratio $\nu_{\mathrm{lt}}^{r}$, transverse Young’s modulus $E_{t}^{r}$ and Poisson’s ratio $\nu_{\mathrm{tt}}^{r}$, and longitudinal shear modulus $G_{\mathrm{lt}}^{r}=G_{\mathrm{tl}}^{r}$ can be obtained from the FEA of the RVE under longitudinal normal, transverse normal, and longitudinal shear loadings, respectively.

The appropriate constraints on the RVE under various loads have been determined by Sun and Vaidya [16]. For longitudinal and transverse normal loading, one-eighth of the RVE ($0\leq x\leq L_{}^{m}/2,\;0\leq y\leq W_{}^{m}/2,\;0\leq z\leq W_{}^{m}/2$) was considered, as shown in Figure 1b. The displacement boundary conditions for the RVE under normal load in the longitudinal x-direction are [17]

(3) $$\begin{array}{c} u_{x}^{\delta }\left(0,y,z\right)=0\\ u_{x}^{m}\left(L^{m}/2,y,z\right)=u_{x}^{*} \end{array}\}\;0\leq y\leq W^{m}/2,\;0\leq z\leq W^{m}/2\;(\delta =m,\;f)$$

(4) $$\begin{array}{c} u_{y}^{\delta }\left(x,0,z\right)=0\\ u_{y}^{m}\left(x,W^{m}/2,z\right)=u_{y}^{0} \end{array}\}\begin{array}{cc} & 0\leq x\leq L^{m}/2,\begin{array}{cc} & 0\leq z\leq W^{m}/2 \end{array} \end{array}\;(\delta =m,\;f)$$

(5) $$\begin{array}{c} u_{z}^{\delta }\left(x,y,0\right)=0\\ u_{z}^{m}\left(x,y,W^{m}/2\right)=u_{z}^{0} \end{array}\}\begin{array}{cc} & 0\leq x\leq L^{m}/2,\begin{array}{cc} & 0\leq y\leq W^{m}/2 \end{array} \end{array}\;(\delta =m,\;f)$$

where $u_{x}^{\delta },\;u_{y}^{\delta }$, and $u_{z}^{\delta }$ are the displacement components in the x-, y-, and *z*- directions, respectively; $u_{x}^{*}$ is the applied uniform displacement in the x-direction; and $u_{y}^{0}$ and $u_{z}^{0}$ are the uniform displacements in the y- and z-directions. The uniform displacements in the y- and z-directions are determined from the condition that resultant stresses at *y* = *W*^m^/2 plane and *z* = *W*^m^/2 plane are zero, respectively. The longitudinal Young’s modulus $E_{l}^{r}=E_{x}^{r}$ and Poisson’s ratio $\nu_{\mathrm{lt}}^{r}=\nu_{xy}^{r}=\nu_{xz}^{r}$ are given by the following equations [16]:

(6) $$E_{x}^{r}=\frac{\sigma_{xx}^{*}}{u_{x}^{*}/\left(L^{m}/2\right)}$$

(7) $$\nu_{xy}^{r}=-\frac{u_{y}^{0}/(W^{m}/2)}{u_{x}^{*}/\left(L^{m}/2\right)}$$

where $\sigma_{xx}^{*}$ is the mechanical mean stress on the $x=L_{}^{m}/2$ plane of the RVE. The mechanical mean stress $\sigma_{xx}^{*}$ is obtained as follows [16,17]

(8) $$\sigma_{xx}^{*}=\frac{\int_{0}^{W^{m}/2}\int_{0}^{W^{m}/2}\sigma_{xx}^{m}\left(L^{m}/2,y,z\right)dydz}{{\left(W^{m}/2\right)}^{2}}$$

Transverse normal loading can be simulated by applying the uniform displacement in the y- or z-direction. An analysis procedure like that used for the case of longitudinal normal loading can be employed for the case of transverse normal loading, and the transverse Young’s modulus $E_{t}^{r}=E_{y}^{r}=E_{z}^{r}$ and Poisson’s ratio $\nu_{\mathrm{tt}}^{r}=\nu_{yz}^{r}$ = $\nu_{zy}^{r}$ can be obtained.

For longitudinal shear loading, a whole RVE $(-L_{}^{m}/2\leq x\leq L_{}^{m}/2,-W_{}^{m}/2\leq y\leq W_{}^{m}/2,-W_{}^{m}/2\leq z\leq W_{}^{m}/2$) was considered. The displacement boundary conditions for the RVE under longitudinal shear load are [17]

(9) $$\begin{array}{c} u_{x}^{m}\left(x,y,-W^{m}/2\right)=0\\ u_{y}^{m}\left(x,y,-W^{m}/2\right)=0\\ u_{z}^{m}\left(x,y,-W^{m}/2\right)=0\\ u_{x}^{m}\left(x,y,W^{m}/2\right)=u_{x}^{*}\\ u_{y}^{m}\left(x,y,W^{m}/2\right)=0\\ u_{z}^{m}\left(x,y,W^{m}/2\right)=0 \end{array}\}\begin{array}{cc} & -L^{m}/2\leq x\leq L^{m}/2,\begin{array}{cc} & -W^{m}/2\leq y\leq W^{m}/2 \end{array} \end{array}$$

In addition, the points on the $x=-L_{}^{m}/2$ and $x=L_{}^{m}/2$ planes with the same y- and z-coordinates need to be constrained to have the same displacements in all three directions as follows [16]:

(10) $$\begin{array}{c} u_{x}^{m}\left(-L^{m}/2,y,z\right)=u_{x}^{m}\left(L^{m}/2,y,z\right)\\ u_{y}^{m}\left(-L^{m}/2,y,z\right)=u_{y}^{m}\left(L^{m}/2,y,z\right)\\ u_{z}^{m}\left(-L^{m}/2,y,z\right)=u_{z}^{m}\left(L^{m}/2,y,z\right) \end{array}\}\begin{array}{cc} & -W^{m}/2\leq y\leq W^{m}/2,\begin{array}{cc} & -W^{m}/2\leq z\leq W^{m}/2 \end{array} \end{array}$$

The longitudinal shear modulus $G_{\mathrm{lt}}^{r\;}=G_{\mathrm{tl}}^{r}=G_{zx}^{r}$ is given by [16]

(11) $$G_{zx}^{r}=\frac{\sigma_{zx}^{*}}{u_{x}^{*}/W^{m}}$$

The mechanical mean stress $\sigma_{zx}^{*}$ is obtained by [16,17]

(12) $$\sigma_{zx}^{*}=\frac{\int_{-W^{m}/2}^{W^{m}/2}\int_{-L^{m}/2}^{L^{m}/2}\sigma_{zx}^{m}\left(x,y,W^{m}/2\right)dxdy}{L^{m}W^{m}}$$

In the analysis, the interface between the CNF and matrix was assumed to be perfectly bonding. The eight-node, three-dimensional solid elements were employed to mesh the RVE. The RVE model under longitudinal and transverse normal loadings consisted of 62,736 nodes and 1400 elements, while the model for longitudinal shear loading had 229,869 nodes and 55,680 elements.

### 2.3. Definition of Randomly Oriented CNF-reinforced Composite

**Figure 2:**
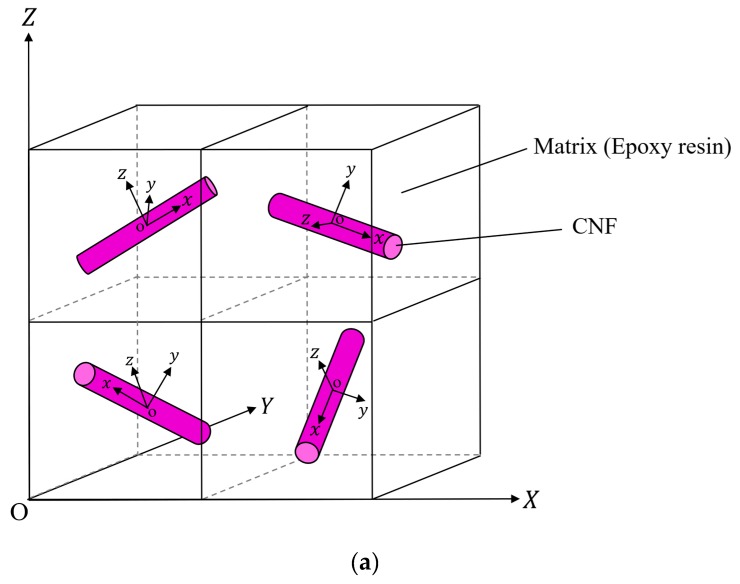

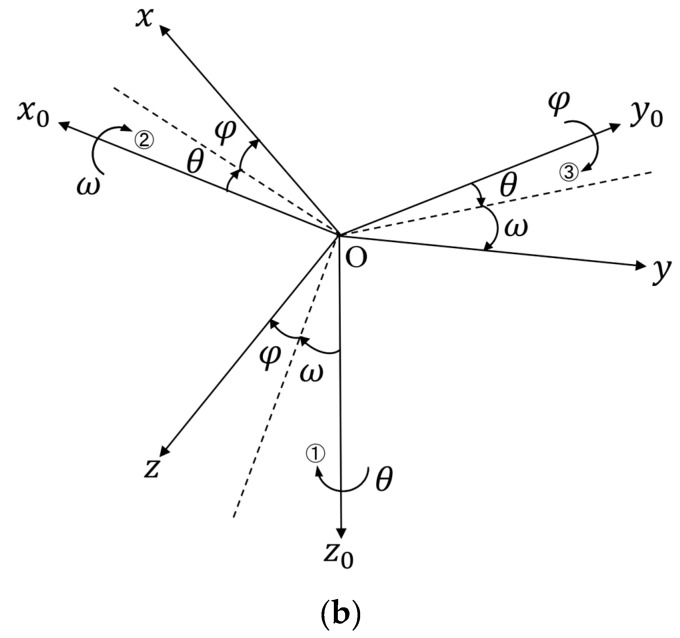
Image of (**a**) the polymer composite with randomly oriented CNF in the FEA and (**b**) the rotation of coordinates.

Figure 2a shows the image of the polymer composite with randomly oriented CNF in the FEA. The global Cartesian coordinate system O-XYZ and the local Cartesian coordinate systems o-xyz for the composite are shown. To create a model of randomly oriented CNF-reinforced polymer composite, the mechanical properties of the RVE denoted by the superscript r were applied to each element, and all local coordinate systems were rotated randomly for the global coordinate system, as shown in Figure 2b, although the geometries of the element and unit cell are different. This random rotation of each element coordinate system was conducted based on the following equation:

(13) $$\left(\begin{array}{c} x\\ y\\ z \end{array}\right)=\left(\begin{array}{ccc} \cos\varphi & 0 & \sin\varphi \\ 0 & 1 & 0\\ -\sin\varphi & 0 & \cos\varphi \end{array}\right)\left(\begin{array}{ccc} 1 & 0 & 0\\ 0 & \cos\omega & -\sin\omega \\ 0 & \sin\omega & \cos\omega \end{array}\right)\left(\begin{array}{ccc} \cos\theta & -\sin\theta & 0\\ \sin\theta & \cos\theta & 0\\ 0 & 0 & 1 \end{array}\right)\left(\begin{array}{c} x_{0}\\ y_{0}\\ z_{0} \end{array}\right)$$

where $\left(x_{0},\;y_{0},\;z_{0}\right)$ represents the initial axes before rotation. The initial orientation of the local coordinate systems was the same as that of the global coordinate system, and ω, φ, and θ are the Euler angles indicating the orientation of the local coordinates (x, y, z) with respect to the global coordinates (X, Y, Z). Furthermore, these were generated from random numbers for each element. By using the above definition, it could be possible to simulate randomly oriented CNF-reinforced polymer composites.

The polymer composite with randomly oriented transversely isotropic RVEs (randomly oriented CNFs) is isotropic material [18]. Hence, the constitutive equation for the composite can be written in the following form:

(14) $$\left(\begin{array}{c} \varepsilon_{XX}^{c}\\ \varepsilon_{YY}^{c}\\ \varepsilon_{ZZ}^{c}\\ 2\varepsilon_{YZ}^{c}\\ 2\varepsilon_{ZX}^{c}\\ 2\varepsilon_{XY}^{c} \end{array}\right)=\left(\begin{array}{cccccc} 1/E^{c} & -\nu^{c}/E^{c} & -\nu^{c}/E^{c} & 0 & 0 & 0\\ -\nu^{c}/E^{c} & 1/E^{c} & -\nu^{c}/E^{c} & 0 & 0 & 0\\ -\nu^{c}/E^{c} & -\nu^{c}/E^{c} & 1/E^{c} & 0 & 0 & 0\\ 0 & 0 & 0 & 1/G^{c} & 0 & 0\\ 0 & 0 & 0 & 0 & 1/G^{c} & 0\\ 0 & 0 & 0 & 0 & 0 & 1/G^{c} \end{array}\right)\left(\begin{array}{c} \sigma_{XX}^{c}\\ \sigma_{YY}^{c}\\ \sigma_{ZZ}^{c}\\ \sigma_{YZ}^{c}\\ \sigma_{ZX}^{c}\\ \sigma_{XY}^{c} \end{array}\right)$$

where the superscript c represents the composite. The mechanical properties of the composites denoted by the superscript c were obtained using the mechanical properties of the RVE denoted by the superscript r.

### 2.4. Finite Element Analysis of Randomly Oriented CNF-reinforced Composite Specimen

**Figure 3:**
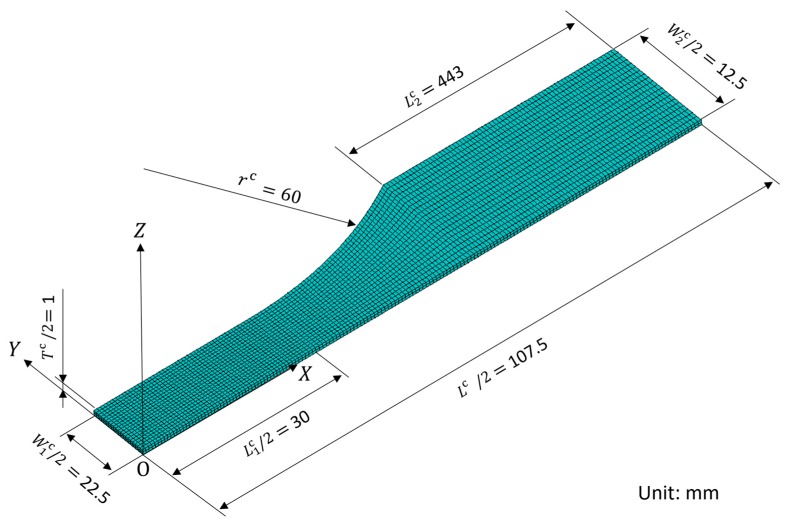
One-eighth finite element model of tensile specimen.

We performed the FEA by using a one-eighth model of a JIS K 7164 tensile specimen, as shown in Figure 3. The global Cartesian coordinate system O-XYZ was employed, and the local coordinate system o-xyz was defined (not shown here) and rotated randomly. $L^{c}$ = 215 mm represents the total length; $L_{1}^{c}$ = 60 mm and $L_{2}^{c}$ = 44.3 mm are the lengths of the reduced section and the grip section, respectively; $W_{1}^{c}$ = 45 mm and $W_{2}^{c}$ = 25 mm are the widths of the reduced section and the grip section, respectively; $T^{c}$ = 2 mm is the total thickness; and $r^{c}$ = 60 mm represents the curvature radius.

The boundary conditions are represented by the following equations:

(15) $$u_{X}^{c}\left(0,\;Y,Z\right)=0$$

(16) $$u_{Z}^{c}\left(X,\;Y,0\right)=0$$

(17) $$u_{X}^{c}\left(L^{c}/2,\;Y,Z\right)=u_{X}^{**}$$

where $u_{X}^{c}$, $u_{Y}^{c},\;u_{Z}^{c}$ represent the displacement components in X-, Y-, and Z- directions, and $u_{X}^{**}$ denotes the applied displacement.

For the finite element model, the eight-node, three-dimensional solid elements were employed. The model consisted of 3141 nodes and 6800 elements.

From the FEA, the stress and strain of the tensile specimens were obtained by averaging these values of the nodes in the reduced section surface ($0\leq X\leq L_{1}^{c}/2,\;0\leq Y\leq W_{1}^{c}/2,Z=T^{c}/2$) because the calculated solutions were different for each node due to the model of randomly oriented CNF-reinforced polymer composites. Additionally, to simulate the randomness of the solutions, an FEA was performed for each three times under the same conditions. Finally, the Young’s modulus $E_{}^{c}$ obtained from the FEA was compared with the experimental results.

## 3. Experimental Procedure

**Figure 4:**
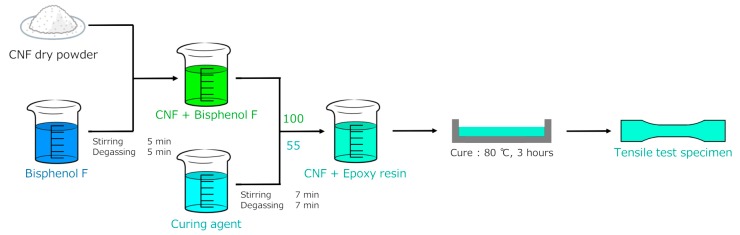
Schematic illustration of the preparation route for CNF/epoxy composite specimen.

In this work, nanocomposites consisting of an epoxy matrix filled with dry CNFs were considered. The diameters of the dry CNFs ranged from 25 to 50 nm, whereas their lengths were between 5 and 50 μm. The processing concept for CNF/epoxy composite is summarized in Figure 4. The fabricated composite samples contained CNF concentrations of 0.73, 2.2, and 3.7 vol %. Neat epoxy samples were also prepared. 

Tensile tests were conducted on the CNF/epoxy composite specimen following JIS K 7164, and the stress–strain curves were measured. The stress was determined by dividing the load by the cross-sectional area of the narrow section. Test specimen dimensions are shown in Figure 3. At least five tests were performed under each condition.

## 4. Results and Discussion

### 4.1. Parameters of Representative Volume Element

The Young’s modulus of the CNF is $E^{f}=$ 138 GPa [7]. The Poisson’s ratio $\nu_{}^{f}$ was assumed to be equal to that of carbon nanotube and hence $\nu_{}^{f}=0.2$ [17]. On the other hand, the Young’s modulus $E^{m}=$ 3.23 GPa and Poisson’s ratio $\nu_{}^{m}=0.34$ of the neat epoxy were obtained from the tensile tests. 

**Figure 5:**
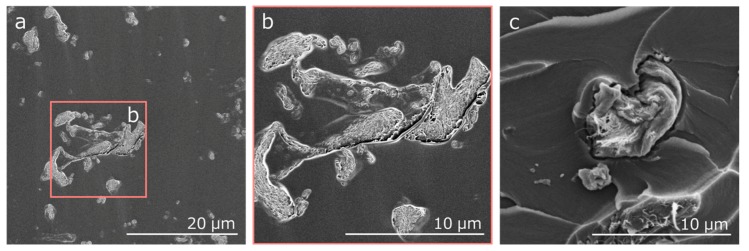
SEM images of (**a**) the surface for the 3.7 vol % CNF/epoxy composite, (**b**) the surface for the 3.7 vol % CNF/epoxy composite (high resolution), and (**c**) the fracture surface for the 0.73 vol % CNF/epoxy composite.

Figure 5 gives the scanning electron microscopy (SEM) images of the surface for the 3.7 vol % and of fracture surface for the 0.73 vol % CNF/epoxy composite specimen. It was found that the agglomerated CNF clusters were almost uniformly dispersed in the epoxy matrix. The fracture of CNF clusters was observed on the fracture surface of CNF/epoxy composites without disentwining. This result indicates that the applied load was transferred to CNF clusters and that CNF clusters contributed to enhance the epoxy resin matrix during the tensile test. The CNF in the epoxy matrix tended to be agglomerated, and the apparent aspect ratio became small. From this image, the aspect ratio $L_{}^{f}$/$d_{}^{f}$ = $L_{}^{m}$/$W_{}^{m}$ = 7.5 was assumed, taking $L_{}^{f}$ and $d_{}^{f}$ as the length and diameter of the agglomerated CNF cluster. For an aspect ratio above 50, the predicted Young’s modulus is almost independent of the aspect ratio [17]. For simplicity here, the agglomerated CNF clusters were assumed to be isotropic and to have the same Young’s modulus and Poisson’s ratio as the CNF.

### 4.2. CNF/epoxy Composite Analysis

**Figure 6:**
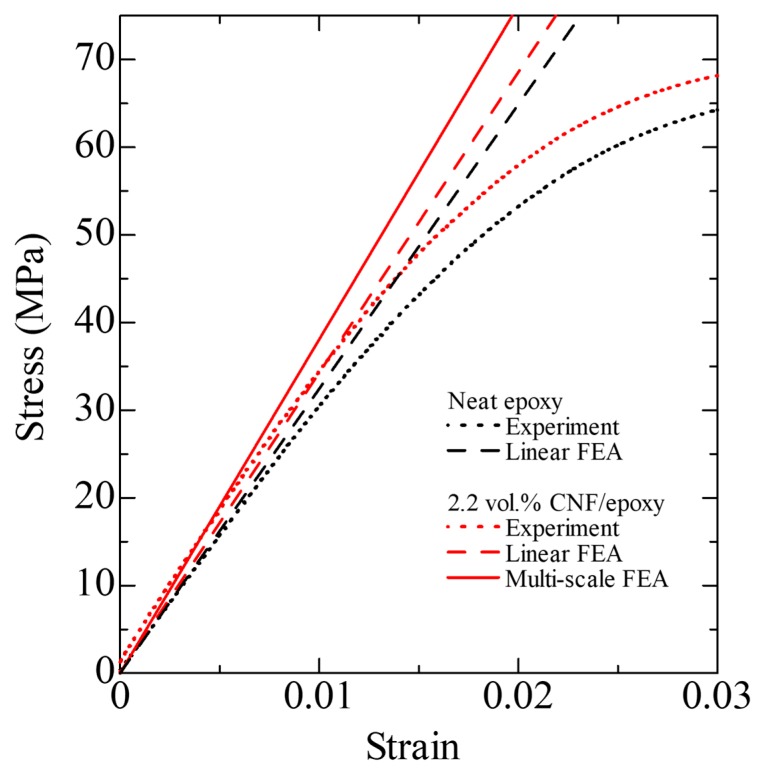
Predicted and experimental stress–strain curves for the neat epoxy and CNF/epoxy composite.

**Figure 7:**
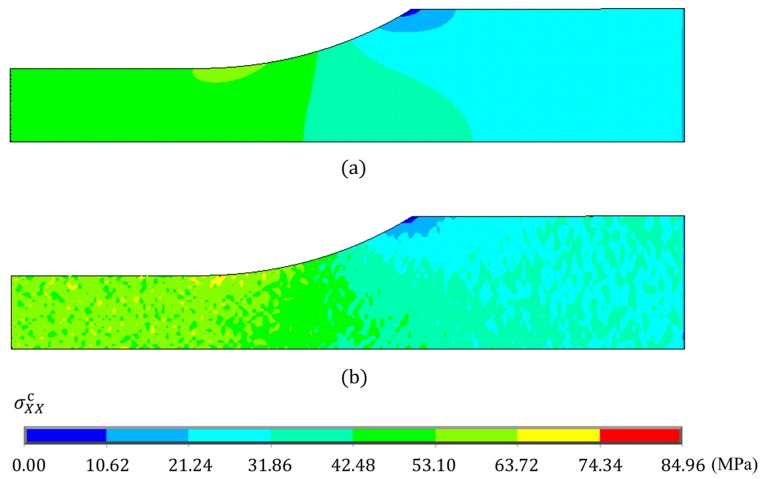
Predicted tensile stress $\sigma_{XX}^{c}$ distributions of randomly oriented CNF/epoxy composite under the tensile strain of 0.015 obtained from (**a**) linear FEA and (**b**) multi-scale FEA. The volume fraction is 2.2 vol %, and the aspect ratio is 7.5.

Figure 6 shows the measured and calculated stresses as a function of the measured strain for the neat epoxy and 2.2 vol % CNF/epoxy composite. Dotted lines indicate the measured data. Dashed lines are the calculated normal stress in the *X*-direction at a chosen point (*X* = *Y* = 0 mm and *Z* = *T*^c^/2 = 1 mm here) from a linear elastic FEA. Each measured curve exhibits the linear initial region, and the calculated curves agree with the measured data very well. In the later loading stage, nonlinearity of the curve exists. This is due to the material nonlinearity. In order to determine the nonlinear stress–strain response numerically, the uniaxial stress–strain behavior, for example, based on Ref. [18] must be represented. More details can be found in our previous works on elastic-plastic finite element modeling [14,19], which is out of scope of the current work. Figure 6 also shows the calculated result for the 2.2 vol % CNF/epoxy composite from the multi-scale FEA (see solid line). It is interesting to note that the stress obtained from the multi-scale FEA was larger than that obtained from the linear FEA. It is anticipated that unexpected high stress is generated in the composite due to the inhomogeneity of the material. The predicted tensile stress $\sigma_{XX}^{c}$ distributions obtained from the (a) linear FEA and (b) multi-scale FEA of the randomly oriented CNF/epoxy composite with the CNF volume fraction $V_{}^{f}$ of 2.2 vol % and the aspect ratio $L^{f}/d^{f}$ = 7.5 under normal strain of 0.015 are shown in Figure 7. From Figure 7a, the uniform stress distribution was generated at the reduced section from the linear FEA. On the other hand, from Figure 7b, it was found that the non-uniform stress distribution was generated at the reduced section from the multi-scale FEA. The results indicate that if the CNFs were randomly dispersed and completely bonded to the epoxy matrix, the stress of the CNF/epoxy composite would be higher. In other words, the linear analysis may underestimate the stress.

**Figure 8:**
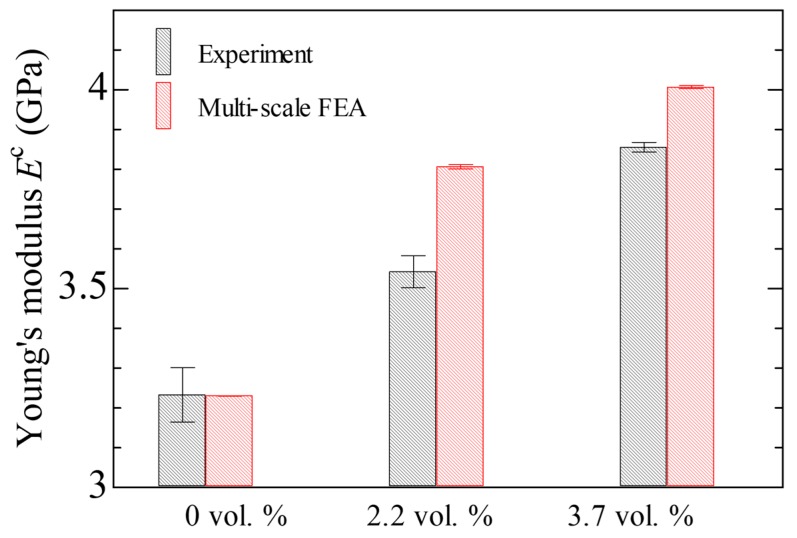
Predicted and experimental Young’s modulus for neat epoxy and CNF/epoxy composites.

Figure 8 shows the measured and predicted Young’s modulus for the neat epoxy, the 2.2 vol % CNF/epoxy composite, and the 3.7 vol % CNF/epoxy composite. The randomness of the solutions was confirmed for each CNF volume fraction in the composites. For the neat epoxy, the predicted Young’s modulus agreed with the measured data. On the other hand, the predicted Young’s moduli for the CNF/epoxy composites were larger than the measured data. Additionally, although the results are not shown here, when a CNF aspect ratio of 50 was used, the predicted Young’s modulus for the 3.7 vol % CNF/epoxy composite became approximately $E_{}^{c}$ = 4.65 GPa. The predicted Young’s modulus $E_{}^{c}$ tends to increase as the CNF volume fraction and aspect ratio increase. This implies that the dispersion of the agglomerated CNF cluster with a high spectral ratio, even in randomly oriented CNF/epoxy composites, increases the Young’s modulus.

Table 1 presents a comparison of the measured Young’s modulus of the present CNF/epoxy nanocomposite with other nanocomposites from the literature. Although dry CNFs were agglomerated in the epoxy matrix, these CNFs seem to tend to increase the Young’s modulus of the epoxy matrix as much as they do other nanomaterials [20,21,22,23], except for aligned multi-walled CNT [24]. On the other hand, the flexural modulus was significantly increased by the extremely low CNF slurry addition [11]. This increment cannot be explained by the theories for discontinuous fiber-reinforced composites. Therefore, it seems that the CNF slurry behaves not as a reinforcing fiber in the epoxy matrix but as an auxiliary agent to enhance the structure of the epoxy molecule.

**Figure 9:**
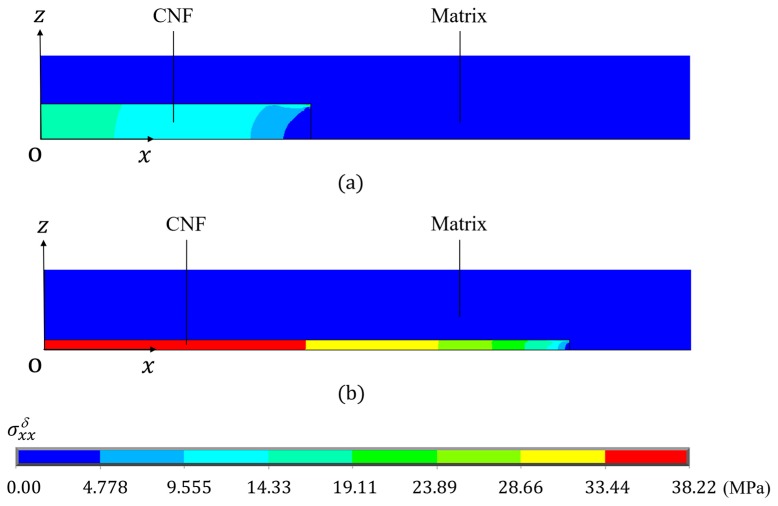
Predicted tensile stress distribution of CNF in RVE with 0.073 vol % under the applied tensile stress of 1 MPa. The aspect ratios are (**a**) 7.5 and (**b**) 50, respectively.

Figure 9 shows the distribution of predicted tensile stress $\sigma_{xx}^{\delta }$ at *y* = 0 plane for the RVE of the composite with the CNF volume fraction *V*^f^ of 3.7 vol % and the aspect ratio $L^{f}/d^{f}$ = 7.5 and 50 under the applied tensile stress of 1 MPa. The stress gradients were confirmed, and the stress increased and became constant toward the center of the CNF. It was found that the stress at the center of the RVE for $L^{f}/d^{f}$ = 50 was larger than that of $L^{f}/d^{f}$ = 7.5.

There are many methods to estimate the overall mechanical and physical properties of composites [17,23,24]. In the present work, we assume the perfect bonding between the CNF and epoxy matrix because of its simplicity and use the three-dimensional finite element method. In fact, a weak boundary exists (see Figure 4). Additional modeling is required for a full interpretation of the mechanical and physical properties of CNF/epoxy composites. The main achievements of this research include the development of a finite element model that can be easily extended to complex problems (e.g., CNF-reinforced polymer composites with interfacial layers, CNF-reinforced polymer composites with slip at interfaces).

## 5. Conclusions

In this paper, we studied the tensile behavior of CNF/epoxy composites by performing multi-scale FEA and tensile tests. It was shown that by increasing CNF content, the Young’s modulus increases. It was also found that agglomeration of CNF decreases the aspect ratio of CNF and decreases the Young’s modulus. A correlation, obtained between the numerical and experimental results for the Young’s moduli of the CNF/epoxy composites under tension, revealed that unexpected high stress is generated in CNF/epoxy composites due to the inhomogeneity, and the inhomogeneity tends to increase the Young’s modulus. The results from this study offer some basic insights into the reinforcing mechanisms in CNF/epoxy composites under tension. These will enable the design of multifunctional nanocomposite materials based on CNF for advanced engineering applications.

All conclusions are based on the numerical predictions for CNF/epoxy composites with perfect bonding between the CNF and epoxy matrix. Some nanocomposites indicate the presence of weak boundaries. This is a challenging research area, and sooner or later, some progress will be made.

## Figures and Tables

**Table 1:** Comparison of the Young’s modulus of epoxy matrix nanocomposites.

Nanomaterial	Content	Modulus (GPa)	Increase Rate (%)	
Dry CNF	0	3.23		This study
	0.73 vol %	3.30	2.1	
	2.2 vol %	3.54	10	
	3.7 vol %	3.85	19	
Silica	0	2.96		[20]
	2.5 vol %	3.20	8.1	
	4.9 vol %	3.42	16	
	7.1 vol %	3.57	21	
	9.6 vol %	3.60	22	
	13.4 vol %	3.85	30	
CNT	0	2.90		[21]
	0.1 wt.%	3.01	3.8	
	0.2 wt.%	3.11	7.2	
	0.3 wt.%	3.26	12	
Aligned CNT	0	2.5		[24]
	4.5 vol %	19	660	
	8.4 vol %	32	1180	
	21.4 vol %	50	1900	
	0	2.69		[22]
Graphene (GP)	4.0 wt.%	2.89	7.4	
Surface-modified GP	4.0 wt.%	3.27	22	
Clay	0	3.53		[23]
	2.0 wt.%	3.58	1.4	
	5.0 wt.%	3.66	3.7	
CNF slurry	0	2.20 *		[11]
	0	2.20 *	26	
	0.25 vol %	3.67 *	67	
	0.74 vol %	2.84 *	29	

* Flexural modulus.
